# Sterilization Procedure for Temperature-Sensitive Hydrogels Loaded with Silver Nanoparticles for Clinical Applications

**DOI:** 10.3390/nano9030380

**Published:** 2019-03-06

**Authors:** Diana Rafael, Fernanda Andrade, Francesc Martinez-Trucharte, Jana Basas, Joaquín Seras-Franzoso, Marta Palau, Xavier Gomis, Marc Pérez-Burgos, Alberto Blanco, Alba López-Fernández, Roberto Vélez, Ibane Abasolo, Màrius Aguirre, Joan Gavaldà, Simó Schwartz

**Affiliations:** 1 Drug Delivery and Targeting Group, Molecular Biology and Biochemistry Research Centre for Nanomedicine (CIBBIM-Nanomedicine), Vall d’Hebron Institut de Recerca (VHIR), Universitat Autònoma de Barcelona (UAB), 08035 Barcelona, Spain; diana.fernandes_de_so@vhir.org (D.R.); francesc.martinez@vhir.org (F.M.-T.); joaquin.seras@vhir.org (J.S.-F.); marcpburgos@gmail.com (M.P.-B.); ibane.abasolo@vhir.org (I.A.); 2 Networking Research Centre for Bioengineering, Biomaterials and Nanomedicine (CIBER-BBN), Instituto de Salud Carlos III, 28029 Madrid, Spain; 3 i3S-Instituto de Investigação e Inovação em Saúde, Universidade do Porto, Rua Alfredo Allen 208, 4200-180 Porto, Portugal; 4 INEB-Instituto Nacional de Engenharia Biomédica, Universidade do Porto, Rua Alfredo Allen 208, 4200-180 Porto, Portugal; 5 Antimicrobial Resistance Laboratory, Vall d’Hebron Research Institute (VHIR), Department of Infectious Diseases, Hospital Universitari Vall d’Hebron, 08035 Barcelona, Spain; jana.basas@vhir.org (J.B.); marta.palau@vhir.org (M.P.); xavier.gomis@vhir.org (X.G.); joan.gavalda@vhir.org (J.G.); 6 Spanish Network for Research in Infectious Diseases (REIPI RD16/0016/0003), Instituto de Salud Carlos III, 28029 Madrid, Spain; 7 Orthopedic Surgery Department, Hospital Universitari Vall d’Hebron, Universitat Autònoma de Barcelona (UAB), 08035 Barcelona, Spain; dr.blancosevilla@gmail.com (A.B.); robertovelez1@gmail.com (R.V.); marius.aguirre@vhir.org (M.A.); 8 Musculoskeletal Tissue Engineering Group, Vall d’Hebron Research Institute (VHIR), Universitat Autònoma de Barcelona (UAB), Passeig de la Vall d’Hebron 129-139, 08035 Barcelona, Spain; alba.lopez@vhir.org

**Keywords:** hydrogels, poloxamer, silver nanoparticles, sterilization, anti-bacterial agents, XDR strains, post-operative infections, chirurgic applications

## Abstract

Hydrogels (HG) have recognized benefits as drug delivery platforms for biomedical applications. Their high sensitivity to sterilization processes is however one of the greatest challenges regarding their clinical translation. Concerning infection diseases, prevention of post-operatory related infections is crucial to ensure appropriate patient recovery and good clinical outcomes. Silver nanoparticles (AgNPs) have shown good antimicrobial properties but sustained release at the right place is required. Thus, we produced and characterized thermo-sensitive HG based on Pluronic^®^ F127 loaded with AgNPs (HG-AgNPs) and their integrity and functionality after sterilization by dry-heat and autoclave methods were carefully assessed. The quality attributes of HG-AgNPs were seriously affected by dry-heat methods but not by autoclaving methods, which allowed to ensure the required sterility. Also, direct sterilization of the final HG-AgNPs product proved more effective than of the raw material, allowing simpler production procedures in non-sterile conditions. The mechanical properties were assessed in *post mortem* rat models and the HG-AgNPs were tested for its antimicrobial properties in vitro using extremely drug-resistant (XDR) clinical strains. The produced HG-AgNPs prove to be versatile, easy produced and cost-effective products, with activity against XDR strains and an adequate gelation time and spreadability features and optimal for in situ biomedical applications.

## 1. Introduction

Hydrogels (HG) are three-dimensional, cross-linked polymer networks that absorb large quantities of water. The use of HG have been highly proposed for biomedical applications, namely drug delivery and tissue engineering [1,2]. An ideal HG should be biocompatible, have an easy production method and be easily tuneable in order to be modulated in terms of mechanical properties, according to the requirements of a desired application. Among the different types of HG, the stimuli-responsive ones are the most attractive for biomedical and tissue engineering applications due to their ability to respond according to surrounding environmental changes like temperature, pH, light, ionic strength and magnetic field [3]. For example, temperature-sensitive HG undergo a phase transition (liquid/sol to gel) above or below a certain temperature called critical solution temperature (CST) and this phenomenon is usually polymer concentration dependent. In the case of HG based in Poloxamers, a polyethylene-propylene glycol copolymer, at low temperatures they are in liquid state (sol) at low temperatures, due to the presence of hydrogen bonds between water and their hydrophilic ethylene oxide chains (PEO). However, when temperature increases, the poloxamers solution suffers a sol-to-gel transition as a consequence of the hydrogen bonds break and the interactions among their hydrophobic propylene oxide chains (PPO). This is called thermoreversible gelation. The non-covalent interactions formed in this stage return to sol state after temperature decreases [4,5,6]. Regarding this, poloxamers are ideal for the production of in situ-forming gels that can be easily administered with surgical-implanted prosthesis or injected into body cavities, in particular when sustained release of encapsulated compounds is envisaged [7].

Further, silver nanoparticles (AgNPs) have been proposed as a reservoir of silver ions for antimicrobial applications [8]. AgNPs have demonstrated higher antibacterial activity compared to free silver ions due to improved protection against inactivation by biological fluids. In fact, their antimicrobial activity comprehends a broad spectrum of pathogens and shows reduced probability to induce bacterial resistance, most likely because of their multilevel antimicrobial effect [9,10]. Furthermore, controlled release of silver ions at effective concentrations for long periods of time, is feasible. However, local continuous release of AgNPs is difficult and hampered because of their pharmacokinetic and biodistribution features [11]. Thus, the encapsulation of AgNPs into Poloxamer-based Hydrogels (HG-AgNPs) could be an interesting strategy to solve post-operative infections (i.e., those associated to orthopaedic, maxillofacial and traumatology manipulations, among other chirurgical techniques) [9,10]. Nonetheless, adequate sterilization is a critical issue to consider for their potential clinical use. Safe methods to ensure HG sterilization that will not alter the mechanical, functional and chemical properties of either polymers or encapsulated material are required. Different aspects like (i) polymers concentration, (ii) AgNPs concentration, (iii) AgNPs size and (iv) sterilization method should be considered in order to achieve an ideal HG with antimicrobial activity and that could serve as vehicle for sustained delivery of AgNPs and other active compounds.

## 2. Materials and Methods

### 2.1. Hydrogel Synthesis

Hydrogels based on different Pluronic^®^ F127 (BASF, Berlin, Germany) ratios were prepared by the cold method. Briefly, an adequate number of polymers were dissolved in an aqueous medium (deionized water or phosphate buffered saline (PBS, pH 7.4, Life Technologies, Alcobendas, Spain) at 4–5 °C to avoid micellization and/or gelation during the process, with constant stirring until a homogenous solution was obtained. pH and osmolality of the HG were adjusted to meet the values required for injectable formulations.

AgNPs were also added to the aqueous media during the dissolution of the polymer to produce AgNPs loaded HG (HG-AgNPs).

For *post mortem* studies, HG were labelled with Gentian violet (Sigma-Aldrich, Madrid, Spain) at a concentration of 0.02% *w*/*v* to allow easier visualization and handling.

Hydrogel was freeze-dried (VirTis BenchTop Freeze-Dryer from SP Scientific., Gardiner, NY, USA) at room temperature during 24 h to allow their characterization by Scanning Electron Microscopy (SEM), as explained below.

### 2.2. Hydrogel Sterilization

Autoclaving (S1000, Matachana, Barcelona, Spain) (15 min at 121 °C), dry-heat (DRYTIME, JP Selecta, Abrera, Spain) (60 min at 140 °C) and filtration (0.2 µm filter) methods were used to perform terminal sterilization of the final HG or to sterilize the polymers before HG production. The effects of sterilization on the physicochemical characteristics of the HG as well as on the integrity and efficacy of the encapsulated AgNPs were assessed.

### 2.3. Hydrogel Characterization

The obtained hydrogels were characterized by different up-to-date techniques: (i) tube inverting test; (ii) gelation time at physiological conditions; (iii) rheological characterization; (iv) microstructure and morphology by Scanning Electron Microscopy (SEM); (v) composition analysis of the polymer network by Electronic Dispersive X-Ray Spectroscopy (EDX); and (vi) determination of reactive oxygen species.

**• Rheological Characterization**

Viscosity measurements have been performed in the Nanoquim Platform Laboratory at Institut de Ciència de Materials de Barcelona (ICMAB-CSIC). Was performed by using a stress-controlled rheometer (HAAKE RheoStress RS600, Thermo Electron Corp., Waltham, MA, USA), equipped with 25 mm parallel plates. A Peltier system (Anton Paar, Graz, Austria) was used for temperature control. The viscous properties of sol phase were studied at constant temperature by means of flow curves at 4, 22 and 37 °C (shear rate from 1 to 100 s^−1^). Samples were poured in the rheometer at the temperature of interest and maintained in quiescent conditions until reaching thermal stability for isothermal testing. Flow curve tests were conducted on F127 solutions with concentrations of 15, 16, 17.5 and 20% *w*/*v*.

**• Tube Inversion Test**

2 mL of HG prepared as previously described at concentrations ranging between 15 and 40% *w*/*v* were put into a glass vial with an inner diameter of 15 mm. The samples were isothermally maintained for 1 h at 4, 22 and 37 °C, for thermal stability. The tube was inverted to allow a visual inspection of the occurrence of phase transition. The sol and the gel were identified as “flow liquid sol” and “no flow solid gel” in 30 s inspection, respectively.

**• Gelation Time**

Sol-gel phase transition behaviour of aqueous Pluronic^®^ F127 solutions was investigated using the tube inverting method. Each solution at a given concentration ranging between 15 and 40% *w*/*v* was prepared following previously reported protocols. A solution volume of 2 mL was put into a glass vial with an inner diameter of 15 mm. Each sample was subjected to a temperature of 37 °C and periodically inverted to confirm the occurrence of phase transition by visual inspection. The sol and the gel were identified as “flow liquid sol” and “no flow solid gel,” respectively. The time necessary to observe a total sol-gel transition was considered as gelation time.

**• Scanning Electron Microscope and Electronic Dispersive X-ray Spectroscopy**

SEM analysis and Electronic Dispersive X-Ray Spectroscopy (EDX) studies were carried out at the Characterization of Soft-Materials Services of the Institut de Ciència de Materials de Barcelona (ICMAB-CSIC, Bellaterra, Spain). Briefly, samples were prepared by fixing the freeze-dried HG to microscope holder, using a conducting carbon strip and covered with a thin platinum layer. Analysis was carried out by means of a scanning electron microscope (SE Detector, 30 kV, high vacuum, low vacuum and environmental SEM mode, ThermoFisher, Barcelona, Spain) working with EDX Link 300 ISIS (Detector Si(Li), 30 kV, low vacuum 10 Pa, resolution 60 eV, ThermoFisher).

**• Determination of Reactive Oxygen Species**

Because degradation of poloxamer by temperature can generate oxygen species that might influence HG biocompatibility, we analyse ROS production from HG after sterilization. Pierce^TM^ Quantitative Peroxide Assay Kit (ThermoFisher), Aqueous Compatible Formulation was used to quantify the amount of radical species present before and after the sterilization process. Standard dilutions of the samples were performed according to the provider’s protocol.

### 2.4. Post Mortem Hydrogel Manipulation Assay

The hydrogels (17.5 and 20% *w*/*v*) were applied *post mortem* onto bone tissue in an ex vivo Sprague-Dawley rat pilot model (*n* = 4) in order to assess the feasibility of the implantation procedure and the gelation time. The surgical procedures were developed in four cadavers that were euthanized for reasons unrelated to the current study. Briefly, a transverse osteotomy fracture was made in the right femur of each animal and the hydrogel was immediately applied surrounding the osteotomy fracture and the gelation time was recorded. Temperature of the dead animals was maintained at approximately 37 °C by using an electric warm pad. The procedure was conducted in compliance with local, National and European legislation (Decret 214/97, Barcelona, Spain; Real Decreto 53/2013, Madrid, Spain; and Directive 2010/63/EU, Bruxelles, Belgium, respectively).

### 2.5. AgNPs Synthesis and Characterization

AgNPs were produced taking into consideration previously reported protocols [12]. Briefly, a sodium citrate (Na_3_C_6_H_5_O_7_) and tannic acid (C_76_H_32_O_46_) solution was heated under vigorous stirring. After boiling, silver nitrate (AgNO_3_) was added to the solution that immediately became bright yellow. The size of the nanoparticles was controlled by the concentration of the tannic acid, increasing the particle size with the increase of tannic acid concentration. Finally, AgNPs were purified by centrifugation and resuspended in Type 1 ultrapure water (18.2 MΩ.cm at 25 °C, Milli-Q^®^, Darmstadt, Germany). Reagents and solvents for AgNPs synthesis were obtained from Sigma-Aldrich, Madrid, Spain. Further, AgNPs mean hydrodynamic diameter (md) and polydispersity index (Pdi) were measured in water by dynamic light scattering (DLS) and zeta potential was assessed by laser Doppler micro-electrophoresis using a NanoZS (Malvern Instruments, Cambridge, UK) with an angle of 173°. AgNPs morphology was assessed by transmission electron microscopy (TEM) at ICMAB-CSIC (Bellaterra, Spain). For that, samples were placed on a grid, treated with uranil acetate and then observed in a JEM-1210 Transmission Electron Microscope (JEOL Ltd., Tokio, Japan) operating at 120 KV.

### 2.6. Antimicrobial Efficacy in Staphylococcus Aureus by Presto Blue^®^ Assay

Hydrogels, AgNPs and HG-loaded with AgNPs produced as described above were challenged with *Staphylococcus aureus* (ATCC-29737) at a final concentration of 0.3 × 10^6^ colony-forming units (CFU)/mL and bacteria viability monitored by Presto Blue^®^ assay (Life Technologies, Madrid, Spain). Briefly, *Staphylococcus aureus* was grown in lysogeny broth (LB) (BD Difco^TM^, Madrid, Spain) O.N. at 37 °C and 300 rpm. Then a 1:100 dilution of the O.N. was used to inoculate a fresh cell culture. Bacteria were incubated at 37 °C and 300 rpm and cell growth was monitored by OD600 until the culture reached the exponential growth phase, at OD600 = 0.5. At this point the cell culture (approx. at 2.5 × 10^6^ CFU/mL) was diluted in PBS (pH 7.4) at 1 × 10^6^ CFU/mL. On the other hand, a 96 well plate containing HG, AgNPs and HG-AgNPs, in both sterilized and non-sterilized formats, were prepared by adding 50 µL of each sample at 20% *w*/*v* HG and 0.25 mg/mL of Ag. Then 50 µL of bacterial suspension were added and thoroughly mixed. In order to avoid excessive desiccation of the HG 50 µL of PBS were also added and the plate incubated O.N at 37 °C. Cell viability was assessed by adding 10 µL of Presto Blue^®^ reagent per well and after incubation at 37 °C for 24 h. Fluorescence was measured in a BioTeK FLx800 plate reader at excitation/ emission wavelengths of 530/25 and 590/20 respectively. All samples were tested in triplicate.

### 2.7. Minimal Inhibitory Concentration (MIC) in Clinical Extensively Drug-Resistant (XDR) Strains

For the in vitro susceptibility studies, 8 clinical isolates obtained from two Spanish hospitals (Vall d’Hebron University Hospital of Barcelona and University Hospital Clinic of Barcelona) were used. This collection included six Gram-negative and two Gram-positive strains. For Gram-negative strains: two XDR clinical isolates of *Pseudomonas aeruginosa* (Pa3; XDR strain harbouring a VIM-2 carbapenemase, only susceptible to colistimethate and isolate disseminated worldwide (ST-235) and Pa1016; XDR strain harbouring an hyperproduction AmpC, OprD inactivation (Q142X), only susceptible to colistimethate and amikacin and isolate disseminated in Spanish hospitals (ST-175)), two XDR clinical isolates of *Klebsiella pneumoniae* (Kp1; producing CTX-M and OXA-48, only susceptible to amikacin, fosfomycin and colistimethate and Kp2; producing CTX-M, NDM, only sensible to fosfomycin and colistimethate) and two XDR clinical isolates of *Acinetobacter baumannii* (AbI1; isolate harbouring a NDM-2 and an OXA-51, only susceptible to colistimethate and tigecycline (ST-103) and Ab4249; isolate harbouring an OXA-51, OXA-24 and hyperproduction of AmpC, only susceptible to amikacin, colistimethate and tigecycline (ST-24)) were used. For Gram-positive strains: two clinical strains of *Staphylococcus epidermidis* (Se14 and Se94) were used. Moreover, four laboratory reference strains were also used: American Type Culture Collection (ATCC) *Pseudomonas aeruginosa* 27853, ATCC E. coli 25922, ATCC *Acinetobacter baumannii* 19606 and ATCC *Staphylococcus epidermidis* 37854. All strains were kept in skim milk at −80 °C in cryogenic vials storage containers. Prior to each experiment, strains were subcultured in Trypticase Soy Agar (TSA, BioMérieux^®^ SA, France) for 24 h at 37 °C.

The minimum inhibitory concentrations (MICs) of HG-AgNPs and sterilized HG-AgNPs that inhibits the growth of the different strains were determined by the broth microdilution method according to the European Committee on Antimicrobial Susceptibility Testing (EUCAST) guidelines [13]. Briefly, Müeller Hinton Broth II (MHBII; Becton Dickinson, Le Pont de Clarx, France) medium was added into each well of a 96-well microtiter plate (Deltalab S.L, Barcelona, Spain) with the exception of the first column in which the two types of HG-AgNPs were added. Then, the treatments were serially diluted and the inocula adjusted to a final concentration of 5 × 10^5^ CFU/mL was added. The amount of silver in the nanoparticles was ranging from 0.125 to 0.00024 mM. A growth control, containing medium and inocula and a sterility control, containing only medium, were also assayed. Finally, plates were incubated at 37 °C for 24 h. The concentration of the HG-AgNPs and sterilized HG-AgNPs needed for inhibiting microbial growth (MIC value) was defined as the lowest concentration of HG-AgNPs that did not allow visible growth after 24 h of incubation. All experiments were performed in triplicate and in the dark.

### 2.8. Statistical Analysis

At least three batches of Hydrogel were produced and characterized. Results were expressed as mean ± SD. For biological studies, at least three replicates, each involving at least two technical replicates, were involved and final results expressed as the mean ± SD. Unpaired Student’s *t*-test was used to determine *p* value. Differences were regarded as statistically significant when *p* value was smaller than 0.05.

## 3. Results

### 3.1. Synthesis and Characterization of Hydrogels

**Figure 1:**
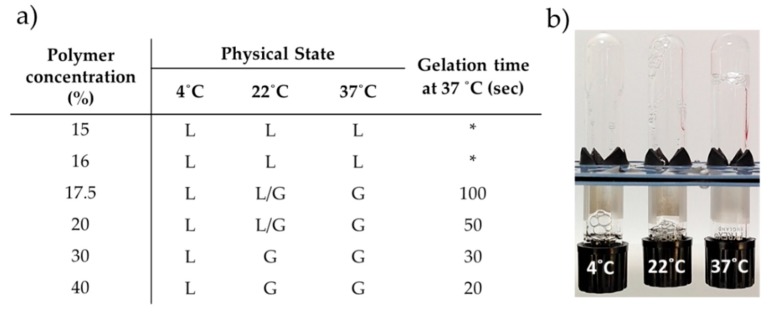
Hydrogel physical characterization. (**a**) Tube inversion test and in vitro gelation time form 22 °C to 37 °C for 2 mL HG volume. (**b**) Tube inversion test photos for the 20% *w*/*v* HG. * No gelation was obtained during the experiment time (24 h). L-Liquid, G-Gel, L/G-more viscous liquid.

**Figure 2:**
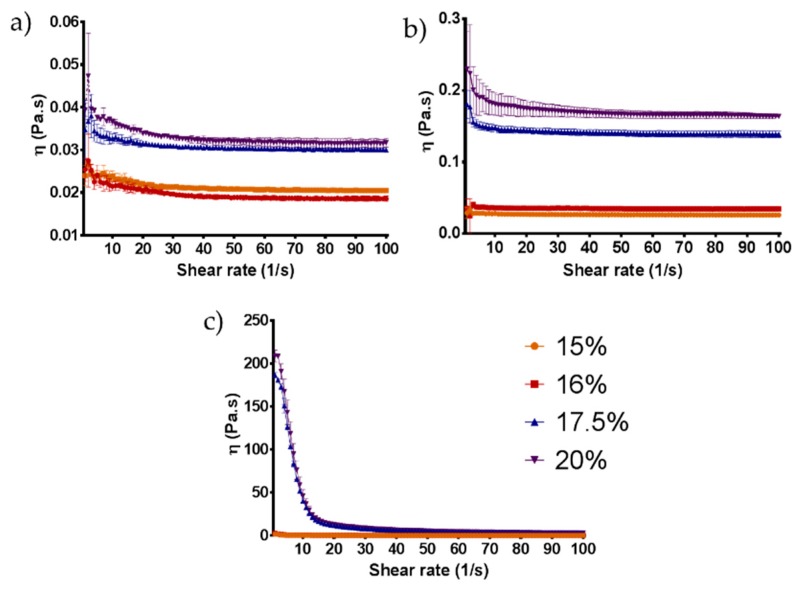
Hydrogel rheological characterization. Viscosity versus shear rate flow curves for different polymer concentrations at 4 °C (**a**), 22 °C (**b**) and 37 °C (**c**).

**Figure 3:**
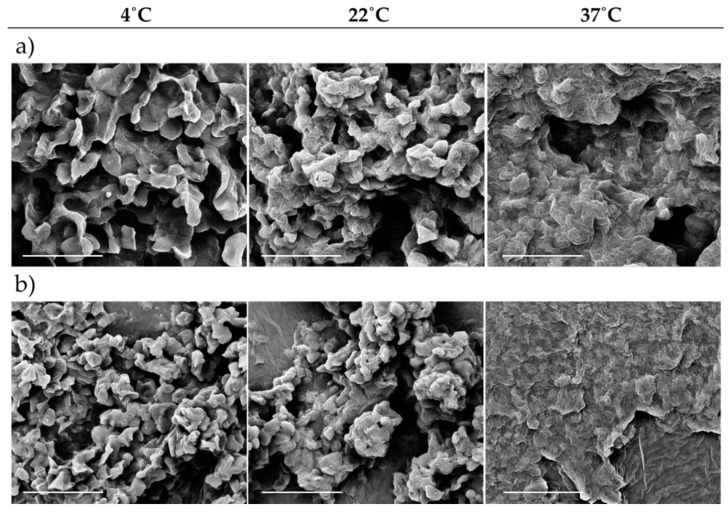
SEM morphologic characterization (scale bar of 10 µm) of 17.5% *w*/*v* (**a**) and 20% *w*/*v* (**b**) HG at different temperatures.

Hydrogels were produced at different polymer concentrations and characterized in terms of rheological behaviour. Hydrogels with 17.5 and 20% *w*/*v* polymer concentration presented the most promising features in terms of sol-gel transition between 22–37 °C, being liquid at 22 °C and hydrogel at 37 °C (Figure 1). Otherwise, no gelation was observed at lower concentrations and at higher concentrations the gel phase occurred at room temperature (Figure 1). Rheological analysis was also performed confirming the results from the tube inversion test. The viscosity of the HG gradually increases with the temperature and polymer concentration (Figure 2). Also, a clear gel phase rheological behaviour was only observed at 37 °C for 17.5 and 20% *w*/*v* HG. Additionally, SEM images taken to lyophilized HG at different temperatures confirmed the HG thermo-reversibility, since a clear morphologic change with the temperature was observed. In Figure 3 is possible to see the disorganization of the polymer fibres at 4 and 22 °C and the formation of a more uniform layer of HG at 37 °C.

### 3.2. Gelation Time In Vitro and Post Mortem

**Figure 4:**
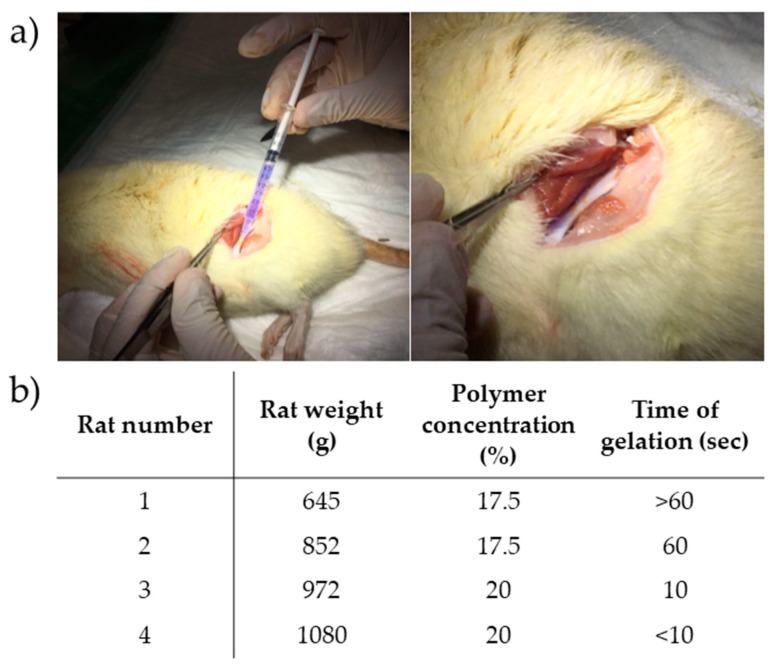
Gelation time *post mortem* in a rat pilot model. (**a**) Gelation allows the HG to remain at the spot of injection *post mortem* (violet circle), (**b**) Rat weight in grams, HG concentration polymer in percentage, and correspondent gelation time in seconds.

Regarding the gelation time assay performed in vitro both 17.5 and 20% *w*/*v* HG suffer a quick sol to gel transition from 22 to 37 °C (less than 100 s) (Figure 1b). Therefore, both concentrations were tested in a post mortem Sprague-Dawley rat pilot model in order to assess which concentration presents better manipulation properties for the implantation procedure. As for in vitro studies, in post mortem experiments 20% *w*/*v* HG presented a lower gelation time (≤10 s) than 17.5% *w*/*v* HG (≥60 s) once applied onto the rat femur, that was maintained at 37 °C with an electrical warm pad (Figure 4). The 20% *w*/*v* HG demonstrated to have ideal conditions of spreadability in situ and due to the fast gelation time was the chosen one to perform further assays.

### 3.3. The Effect of Sterilization Process in the Polymer and in the HG

**Figure 5:**
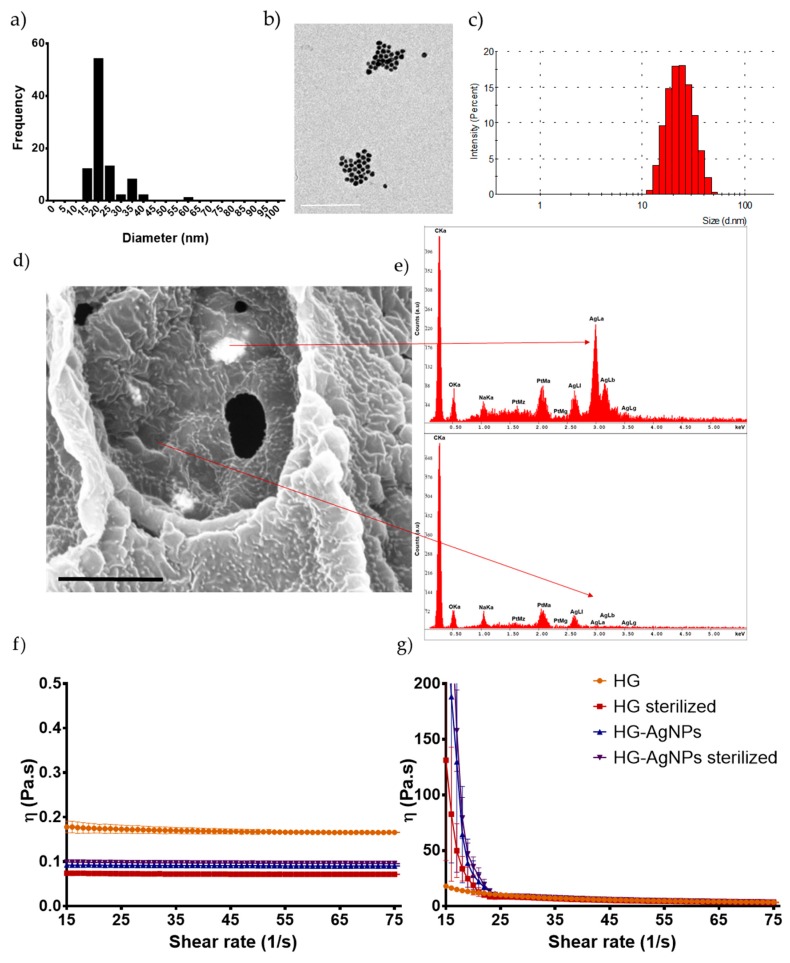
AgNPs and HG-AgNPs characterization (**a**) Histogram of frequency size distribution of AgNPs (nm) determined by TEM (scale bar 200 nm), (**b**) AgNPs TEM micrographs, (**c**) Histogram of size distribution by intensity of AgNPs (nm) determined by DLS (**d**) AgNPs incorporation into 20% *w*/*v* HG visualized by SEM (scale bar 2 µm), (**e**) EDX spectra of the AgNPs incorporated into the HG (upper graphic where is visible a strong peak of Ag) and HG area without AgNPs (lower graphic where Ag peak is almost inexistent), and (**f**) Viscosity versus shear rate flow curves for different 20% *w*/*v* HG samples at 22 °C and (**g**) at 37 °C. Results expressed as mean ± SD, *n* = 3.

The HG and the polymer were sterilized by two conventional methods of sterilization used in the pharmaceutical industry, the autoclave and the dry-heat methods. It was also tested the terminal sterilization of HG by filtration but this technique was discharged from further analysis due to the difficulties imposed by the polymer solution viscosity. The effect of sterilization was assessed in terms of tube inversion test, gelation time and formation of reactive oxygen species. It was observed that both the polymer and the HG are more sensitive to sterilization by dry-heat, since it loses the gelation properties. On the other hand, sterilization by autoclave did not affect significantly differences the gelation properties HG (Table 1 and Table 2). Regarding the polymer, autoclaving slightly impact on the gelation properties as observed by an increase in the gelation time (Table 2). Regarding rheological analysis, is possible to observe an increase in the viscosity of the HG at 37 °C after sterilization by autoclave (Figure 5g).

However, regarding the formation of reactive oxygen species, an important difference between the two methods of sterilization was observed. Table 3 show significantly higher values of peroxides for the dry-heated samples, while lower peroxides formation was detected with the autoclave method when compared with the control. Also, sterilization of the polymer prior the HG formation lead to a high formation of peroxides.

### 3.4. Silver Nanoparticles Production, Characterization and Their Incorporation into 20% w/v HG

AgNPs were prepared following a kinetically seeded-growth approach via the reduction of silver nitrate with sodium citrate and tannic acid, as reducing agents. The obtained particles presented a spherical shape, low polydispersity (0.098 ± 0.03) and a mean diameter of 20.06 ± 7.08 nm measured by TEM and 22.25 ± 0.16 nm measured by DLS (Figure 5a–c). AgNPs were incorporated into 20% *w*/*v* HG obtaining a proper incorporation and distribution of the AgNPs within the hydrogel as observed by SEM and EDX (Figure 5d–e).

The presence of AgNPs do not affect the gelation behaviour when compared with the sterilized unloaded HG (Table 1 and Table 2); however, an increase in the HG viscosity could be observed for 37 °C, while no significant differences where observed at 22 °C (Figure 5f–g). The incorporation of AgNPs to the HG decreased the levels of peroxides formation, possibly due to antioxidant properties of silver (Table 3).

### 3.5. Sterilized Versus Non-Sterilized HG Efficacy

**Figure 6:**
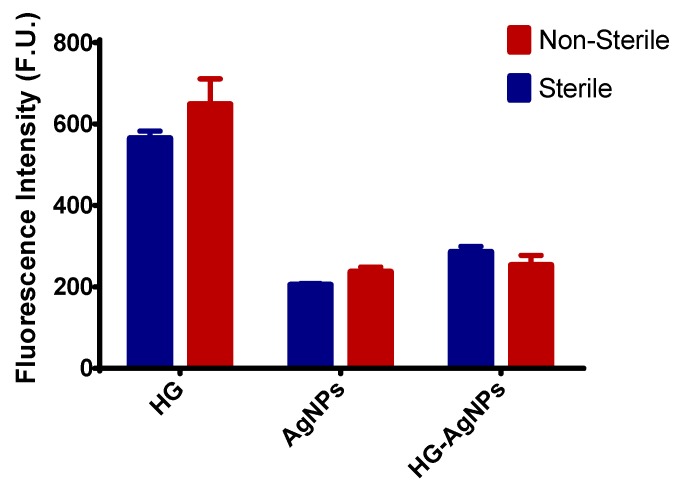
Antibacterial activity of sterilized by autoclave and non-sterilized AgNPs, 20% *w*/*v* HG and HG-AgNPs against *Staphylococcus aureus*. Results are expressed as mean ± SD, *n* = 3.

The antimicrobial efficacy of sterilized and non-sterilized HG as well as of AgNPs were tested in different bacterial strains trough two different techniques. First, as a preliminary assay, the efficacy was tested in a common laboratory strain of reference (*Staphylococcus aureus*) via a viability test using the Presto Blue^®^ reagent. Second, the anti-bacterial effect was also assessed in clinical strains isolated form patients from different Spanish hospitals determining the susceptibility by fluorescence intensity and the Minimal Inhibitory Concentration (MIC) for each isolate (Figure 6 and Table 4).

#### 3.5.1. Efficacy in a Conventional Laboratory Strain: Staphylococcus Aureus

The efficacy assay in Staphylococcus aureus showed that HG-AgNPs have similar antibacterial activity as free AgNPs, presenting both a significant reduction of CFU/mL when compared with the empty HG (*p* < 0.001). In addition, no statistically significant differences (*p* > 0.05) between the activity of sterilized and non-sterilized HG was observed. Thus, the activity of both, HG-AgNPs and AgNPs, is not affected during the sterilization process.

#### 3.5.2. Susceptibility Test in Clinical Extremely Drug-Resistant Strains

AgNPs in vitro susceptibility tests were performed in four clinical strains originally isolated from patients at different Spanish hospitals: two XDR clinical isolates of *Pseudomonas aeruginosa* and two *Staphylococcus epidermidis* clinical strains. ATCC *Pseudomonas aeruginosa* 27853 and ATCC *Staphylococcus aureus* 29213 were used as quality controls. After 24 h incubation at 37 °C with the different strains, AgNPs efficacy was defined as the lowest concentration of AgNPs with which no turbidity was detected in the bacterial culture. The results of MIC susceptibility studies of AgNPs show that the clinical isolates of *Pseudomonas aeruginosa* and *Staphylococcus epidermidis* are susceptible at the tested concentrations (Table S1).

Regarding HG-AgNPs, the results of the susceptibility studies (Table 4) are in accordance with the previous assay. The *Pseudomonas aeruginosa* and the *Staphylococcus epidermidis* isolates used in our study were susceptible to the two types of HG-AgNPs tested (MIC concentration of 0.125 mM in all of them). In addition, no differences were observed between the activities of sterilized or not sterilized HG-AgNPs against the different strains, meaning that sterilization did not have any effect on the antimicrobial properties of the AG-AgNPs. In contrast, for *Klebsiella pneumoniae* and *Acinetobacter baumannii* isolates, no efficacy was observed in any case.

## 4. Discussion

The benefits of using HG for different biomedical applications are widely accepted. However, for its proper clinical applicability in areas such as in orthopaedic surgery for filling bone defects or when spreading HG over a prosthesis is required, the HG must present a sol-gel phase transition below 37 °C, ideally being liquid at room temperature and suffering a quick gelation at body temperature. To reach the aimed properties, different polymer ratios were screened and the mechanical and rheological behaviour assessed. HG of 17.5 and 20% *w*/*v* of polymer presented the best features, since lower concentrations were liquid at 37 °C and higher concentrations suffered gelation below 22 °C (Figure 1). The thermos-sensitivity of the HG observed by the tube inversion test and the gelation time were corroborated by rheological assessment, where it was possible to observe an increased viscosity of the HG proportional to the concentration of the polymer and temperature (Figure 2). The obtained results agree with previous reports of thermos-sensitivity of Pluronic^®^-based HG [14,15,16,17]. The effects of temperature on HG mechanical properties can be also confirmed by alterations on its morphology and microstructure, as observed by SEM, passing from a more disorganized (4–22 °C) to a more compact and organized structure (37 °C) (Figure 3).

To study the feasibility of the HG regarding handling and application in clinical practice, a *post mortem* study was performed using *post mortem* femur exposed rats maintained at 37 °C with electrical warm pads (Figure 4). It was possible to observe that 20% *w*/*v* HG undergoes fast phase transition (≤10 s) but maintains the desired handling properties, allowing proper spreadability of the HG. Thus, 20% *w*/*v* HG was chosen for further development. The main advantage of the proposed HG, when compared with commercial available products [18], is its ready-to-use presentation, not requiring any previous preparation step before application, which facilitates its translation to the clinical practice.

Sterilization of composite biomaterials is crucial for their use in the clinical setting to ensure patient safety. Particularly, in wound healing, surgery/prosthesis and with internal medical devices. However, most HG are sensitive to conventional sterilization methods, suffering severe physicochemical alterations that impact on their mechanical properties and biocompatibility and also, in the efficacy of the loaded therapeutic compounds [19]. There are different possible approaches to produce sterile HG including pre-processing sterilization of the polymers and components and production under sterile conditions and terminal/postprocessing sterilization of the hydrogel using different techniques including steam heat (autoclave), dry-heat, gamma radiation and ethylene oxide, among others.

In this work we tested the pre-processing and terminal sterilization of HG using autoclave and dry-heat of polymers and HG, respectively. Sterilization by filtration did not proceed to further analysis since an efficient procedure was hampered by the polymer solution viscosity. Pre-processing sterilization of the polymers by both techniques has shown to be inadequate because it affected the mechanical properties of the HG, as observed by the alteration of the phase transition temperature (Table 1 and Table 2). Moreover, a significant increase of oxygen reactive species was also observed especially for dry-heat sterilization, which correlated with a reduction of the biocompatibility of the HG (Table 3). On the other hand, no significant differences on the rheological behaviour was observed in the terminal sterilization of the HG by autoclaving (Table 1 and Table 2 and Figure 5f–g). Degradation of poloxamer with temperature occurs mainly by cleavage (possibly by the ether bond) and oxidation with consequent formation of formate and acetate esters and respective aldehydes and acids, as well as peroxy species [20,21]. The differences observed between the pre-processing and terminal sterilization can be due to the 3D conformation of the hydrogel with PPO fragments and the ether bond being more protected and, also, by the hydration of the polymer since the oxidation of Pluronic^®^ can be delayed when dissolved in water [21]. Regarding oxygen reactive species, higher amount of hydrogen peroxide was formed when HG were sterilized by dry-heat, which could be the consequence of higher time and temperature exposition of the product. Based on the results of rheological behaviour and oxygen reactive species generation, terminal steam heat was chosen as method for HG sterilization. Terminal sterilization of a product is highly beneficial for the scale-up production at industrial level since it avoids the necessity of production under sterile conditions, which requires special conditions and infrastructures and involves higher costs.

Postoperative infections are a significant source of preventable perioperative morbidity and mortality, being a burden for patients’ quality of life and health care systems [22,23]. Prophylactic antibiotics have been used to prevent nosocomial infections but their effects are often impaired by the development of highly resistance bacterial strains [24,25,26]. To circumvent antibiotic resistance and provide postoperative infection prophylaxis, AgNPs were loaded within the HG as silver ions have strong antimicrobial activity, even against most resistant strains [27,28]. Spherical and monodisperse AgNPs of around 22 nm were synthesized (Figure 5a–c) and homogeneously dispersed in the HG as observed by SEM-EDX (Figure 5d–e). The incorporation of AgNPs, slightly increase the viscosity of the HG (Figure 5f–g) but did not significantly alter the mechanical properties and the phase transition behaviour (Table 1 and Table 2). Moreover, as for the plain HG, sterilization by autoclave did not interfere with the properties of the HG-AgNPs (Figure 5f–g). Regarding the formation of oxygen reactive species, AgNPs incorporation into the HG seems to decrease the levels of H_2_O_2_. This could be explained by the antioxidant and radical scavenging capacity of AgNPs [29,30], which seems to balance the effects of sterilization on the reactive species formation.

Regarding the antimicrobial activity of AgNPs and HG-AgNPs, a pilot study was performed against *Staphylococcus aureus* (Figure 6). The incorporation of AgNPs into the HG slightly decreased the antibacterial activity of AgNPs, possibly due to a slower release of silver ions from the HG during the time of the experiment, thus being less available to exert its therapeutic effect. Facing clinical applications, a sustained release of AgNPs and silver ions from the HG would have a strong positive perspective because it might allow prolonged prophylactic effects over time. The efficacy of the sterilized and non-sterilized HG-AgNPs was also studied on laboratory reference strains and clinical isolates of XDR *Pseudomonas aeruginosa*, *Klebsiella pneumoniae*, *Acinetobacter baumannii* and *Staphylococcus epidermidis* isolates (Table 4). It was possible to observe susceptibility of all *Pseudomonas aeruginosa* and the *Staphylococcus epidermidis* isolates to both sterilized and non-sterilized HG-AgNPs. No effects were observed against *Klebsiella pneumoniae* and *Acinetobacter baumannii*, in any case. As for *Staphylococcus aureus*, an increase of the MIC was observed when AgNPs were loaded in the HG. Most importantly, terminal sterilization by autoclave of HG-AgNPs, did not interfere its antibacterial activity (Table 4), thus being a useful technique for the sterilization of the final product.

## 5. Conclusions

Current available commercial treatments used for postoperative infection and biofilm prevention require product preparation in the operating room and addition of antimicrobial compounds to the formulation. The proposed HG-AgNPs present adequate handling and applicability properties being an easy-to-apply product that would shorten surgery time and reduce risk of infection due to prolonged wound and prosthesis exposure.

Related to over and misuse of antibiotics, there is an increasing worrying pace of antibiotic resistance. One of the most important aspects of HG-AgNPs is their antimicrobial antibiotic-free property that does not promote antibiotic resistance. Moreover, it exerts strong antibacterial activity against some resistant isolates, thus being a promising product for a broad prophylaxis of postoperative infections. Furthermore, being a product of easy production and terminal sterilization by autoclave, a fast translation of the product as Medical Device to the clinical practice is foreseen.

## Figures and Tables

**Table 1:** Effects of sterilization and temperature in 20% *w*/*v* HG physical state (sample volume: 2 mL, *n* = 3).

Temperature (°C)	Control HG	Dry-Heat		Autoclave		
		Polymer	HG	Polymer	HG	HG-AgNPs
4	L	L	L	L	L	L
22	L	L	L	L	L	L
37	G	L	L	G	G	G

L-Liquid/sol phase; G-Gel phase.

**Table 2:** Effects of sterilization and starting temperature in the 20% *w*/*v* HG gelation time at 37 °C. (sample volume: 2 mL, results expressed in seconds as mean ± SD, *n* = 3).

Temperature (°C)	Control HG	Dry-Heat		Autoclave		
		Polymer	HG	Polymer	HG	HG-AgNPs
4	64.0 ± 1.73	*	*	80.0 ± 7.1	67.5 ± 3.5	69.6 ± 10.1
22	26.3 ± 1.52	*	*	37.0 ± 1.41	23.4 ± 4.0	33.0 ± 2.64

* No gelation observed during the experiment time (2 min). Control HG–non-sterilized.

**Table 3:** Effects of Sterilization in the 20% *w*/*v* HG Reactive oxygen species formation (results expressed as mean ± SD, *n* ≥ 3).

**[H_2_O_2_] (μM)**	**Control HG**	**Dry-Heat**		**Autoclave**		
		**Polymer**	**HG**	**Polymer**	**HG**	**HG-AgNPs**
	3.9 ± 2.0	>65	>65	31.2 ± 4.1	18.4 ± 5.3	6.4 ± 3.7

**Table 4:** In vitro susceptibility of sterilized and non-sterilized HG-AgNPs against *Pseudomonas aeruginosa*, *Klebsiella pneumoniae*, *Acinetobacter baumannii* and *Staphylococcus epidermidis* isolates.

	MIC (mM)	
Strain	HG-AgNP	Sterilized HG-AgNP
Pa3	0.125	0.125
Pa1016	0.125	0.125
ATCC Pa 27853	0.125	0.125
Kp1	>0.125	>0.125
Kp2	>0.125	>0.125
ATCC Ec 25922	>0.125	>0.125
AbI1	>0.125	>0.125
Ab4249	>0.125	>0.125
ATCC Ab 19606	>0.125	>0.125
Se14	0.125	0.125
Se94	0.125	0.125
ATCC Se 37854	0.125	0.125

MIC, minimum inhibitory concentration (in mM); HG-AgNPs, non-sterilized hydrogel with silver nanoparticles; Pa, *Pseudomonas aeruginosa*; Kp, *Klebsiella pneumoniae*; Ab, *Acinetobacter baumannii*; Se, *Staphylococcus epidermidis*.

## Supplementary Materials

The following are available online at https://www.mdpi.com/2079-4991/9/3/380/s1, Table S1: In vitro susceptibility of AgNPs against *Pseudomonas aeruginosa*, *Staphylococcus aureus* and *Staphylococcus epidermidis* isolates.
