# Supplementary material for: Sterilization Procedure for Temperature-Sensitive Hydrogels Loaded with Silver Nanoparticles for Clinical Applications
Source: Nanomaterials (Basel). 2019 Mar 6;9(3):380. doi: 10.3390/nano9030380 (PMC6474025; doi:10.3390/nano9030380)
Supplement: Supplementary file 1 [file nanomaterials-09-00380-s001.pdf]

Sterilization Procedure for Temperature-Sensitive Hydrogels Loaded with Silver Nanoparticles for Clinical Applications

Table S1. In vitro susceptibility of AgNPs against Pseudomonas aeruginosa, Staphylococcus aureus and Staphylococcus epidermidis isolates.

	**MIC (mM)**
Strain	AgNPs
ATCC Pa 27853	0.06
Pa3	0.06
Pa1016	0.125
ATCC Sa 29213	>0.125
MSSA 80004	>0.125
MSSA 170006	>0.125
SE14	0.06
SE94	0.06
